# Stroke survivors and their families receive information and support on an individual basis from an online forum: descriptive analysis of a population of 2348 patients and qualitative study of a sample of participants

**DOI:** 10.1136/bmjopen-2015-010501

**Published:** 2016-04-05

**Authors:** Anna De Simoni, Andrew Shanks, Chantal Balasooriya-Smeekens, Jonathan Mant

**Affiliations:** 1 Centre for Primary Care and Public Health, Barts and The London School of Medicine and Dentistry, London, UK; 2 Department of Primary Care Clinical Sciences, School of Health and Population Sciences, University of Birmingham, Birmingham, UK; 3 Primary Care Unit, Department of Public Health, University of Cambridge School of Clinical Medicine, Cambridge, UK

**Keywords:** online forum, carers, unmet needs, TIA, stroke survivors

## Abstract

**Objective:**

To describe the characteristics of participants of an online stroke forum, their reasons for posting in the forum and whether responses addressed users' needs.

**Methods:**

Descriptive analysis of the population of 2004–2011 archives of Talkstroke, the online forum of the Stroke Association, and comparison with patients admitted to hospital with stroke (Sentinel Stroke National Audit Programme, SSNAP). Thematic analysis of posts from a sample of 59 participants representative of age at stroke and sex.

**Settings:**

UK.

**Main outcome measures:**

Characteristics of participants: age, sex, survivor versus patient by third party, side of stroke (R, L), social class; (from the sample of 59 participants): level of disability, stroke type, classification of users' intents for writing a post in the forum, quantification of needs addressed by the forum, topics of discussion.

**Participants:**

2348 participants (957 stroke survivors, 1391 patients with stroke talked about by third party).

**Results:**

Patients of both sexes and from a wide range of ages at stroke (0 to 95 years) and degrees of disability were represented in the forum, although younger than the UK stroke population (mean age 52 years vs 77 years in SSNAP). Analysis of 841 posts showed that the main users' intents for writing in the forum were requests/offers of information and support (58%) and sharing own experiences of stroke (35%). Most information needs were around stroke-related physical impairments, understanding the cause of stroke and the potential for recovery. Up to 95% of the users' intents were met by the replies received.

**Conclusions:**

Patients' needs expressed in the online forum confirm and widen the evidence from traditional research studies, showing that such forums are a potential resource for studying needs in this population. The forum provided an opportunity for patients and families to give and receive advice and social support.

**Strengths and limitations of this study**

- This is the first study to investigate the characteristics of people with stroke who take part in an online forum, the use they make of it in relation to their condition, and to show the potential value of the emotional and informational support that online forums provide to patients with stroke and their carers.
- The strength of the study lays in the descriptive analysis of the entire population of 2348 forum participants, underpinning the validity of results obtained from analysing the forum archives.
- Forum participants were younger compared with the population of patients with stroke.
- The analysis of the forum was limited by the time users were active in the forum, by the form moderation process, and by the lack of assessment of the authenticity of the forum content.

## Introduction

Unmet needs have been reported by stroke survivors across a range of clinical domains, including need for information and aspects of social participation.^1–3^

Greater social support has been linked to better health outcomes.^4–13^ In chronic diseases, higher social support, most commonly measured as the amount of practical and emotional support that patients perceive, has been linked with better self-management behaviour,^14^ ^15^ improved disease control^16^ and lower mortality risk.^17–21^ Therefore providing social support to patients can improve health outcomes after stroke.

Positive effects have been noted for the person receiving and the person providing support.^22^

While support from healthcare professionals, family and friends is important during post-stroke recovery,^23^ peer support, based on shared experience, may be helpful in decreasing feelings of isolation and fear. A study of local stroke groups^24^ found that support groups helped stroke survivors understand their stroke in a way that health professionals did not (ie, professionals were perceived as not adequately understanding stroke and how patients could cope with recovery), decreased feelings of loneliness or isolation, played a role in overcoming depression, provided opportunity for discussion that was different from speaking to family or friends, changed their understanding of the timeframe for improvement and recovery, and inspired a desire to give back and help others.

Compared with local community stroke groups, the Internet is available ubiquitously, 24 h a day and at a low cost per participant. Online forums allow asynchronous interactions, through which participants can join in the discussions at their own convenience. The potential value of the emotional and informational support that online forums provide has been reported in other patients groups.^25–28^ The number of users and data content of Internet forums can be large, and analysis using traditional research methodologies can be challenging.^29^ Previous analyses of Internet forums have been based on a limited number of participants,^30^ or from collections of posts from several different websites' forums where the user population is not characterised.^31^

One third of stroke survivors have difficulty with communication, and half are dependent on others for daily activities due to stroke-related disabilities.^32–34^ Although there is evidence that people with declared disability are less online compared to those without disability (63% vs 85% in the UK)^35^ and that patients with stroke have difficulties using the Internet,^36^ it is not known whether communications and support through online services is effectively only open to a subgroup of the stroke survivor population, for example, to young, less disabled and computer literate stroke survivors or whether indeed it widens access to support and information since it can all be accessed from the home.

The aim of this study was to evaluate the outreach of an online forum, the intents of users for posting in the forum and whether responses addressed users' needs.

## Methods

This study was conducted on the archives of the Talkstroke forum, a moderated online forum containing posts dated from 2004 to 2011. The Stroke Association has given permission to analyse the archives for research purposes. The database was in .csv file format and included posts, usernames, participants and number of replies per post. To maintain anonymity and protect confidentiality, user profiles were not included (see box 1). Information on the user population was extracted by reading subsequent posts of each user.

Box 1 **Ethical considerations**

▸ There is consensus that Internet data that are freely and publicly accessible can be used for research without prior ethical approval.^37^ ^38^ On this premise, data taken from Internet have been widely used for research purposes. Nevertheless, Internet based research raises ethical questions pertaining to privacy and informed consent.

▸ The subscription to the forum required participants to consent that their posts were public and messages could be accessed online without registration. Consent to use the archive for research purposes was received from the communication officer and the manager of the Talkstroke forum. Given the large number (>2500) of participants and timespan of the data (2004–2011), contacting each single participant to request consent would have been impractical.

▸ Intrusiveness, potential for harm and perception of forum as public or private have been identified as key issues to consider regarding the ethical use of internet data.^39^

▸ The analysis on the forum is classified as passive analysis with low intrusiveness, that is, analysis of information patterns and interactions on discussion groups of which researchers have not been part. The high number (>2500) of users of the community itself is likely to have reinforced to participants that their posts were public. Furthermore, the forum included tens of participants identifying themselves as researchers, asking questions with overt research purposes and receiving replies.

▸ To ensure confidentiality and protect anonymity of participants, we have avoided reporting verbatim quotes, as these could be tracked back to forum usernames using special search engines for archived webpages. Quotes in table 1 have instead been reported as third person descriptions.

**Table 1:** Example of forum discussion, classification of users' intents for writing in the forum and of judgments made on whether the replying intent matched the requesting one (codes used: yes, no, unsure). Quotes are described in third person to protect privacy of participants

User	User's quotes	User's intents	Match
**Requesting user** *(Woman, 46 years, age at stroke 46)*	**A woman was asking other forum users whether cold weather was making stroke symptoms worse**	**Asking** **information**	
** **	**She found that when the weather was colder her speech was slurred more, and her right side weakness and tiredness were worse.**	**Sharing own story**	
Replying user 1 *(Woman, 54 years, age at stroke 46)*	A woman replied that she was the same, and that her physiotherapist told her years earlier that this was down to ‘loss of sensory perception’ and in her case she had lost all her left side.	Providing information	Yes
	She was nearly always cold except in very high temperatures, wearing coat or fleece throughout the summer and full length ‘tubigrip’ on her stroke arm to keep warm.	Sharing own story	Yes
	Her physiotherapist said the tubigrip was also providing ‘sensory perception’ to brain (ie, reminding her brain that her arm was there).	Providing information	Yes
Replying user 2 *(Woman, age and age at stroke not stated)*	A woman wrote back that she also had the same symptoms, she found that her body was not as strong in the cold weather.	Providing information	Yes
	She was dropping things more and had to really concentrate when walking. She had the central heating on full blast so any visitors had to peel off their layers, they were nearly passing out in the heat! She had always felt the cold much more since her stroke.	Sharing own story	Yes
Replying user 3 *(Man, age and age at stroke not stated)*	A man replied that cold weather was affecting him, too.	Providing information	Yes
	He had a holiday in Greece in October, and he walked all over the place. The day he got back to UK he couldn't walk to his local shop and back. He concluded by saying ‘It is not just you!!’	Sharing own story	Yes
Requesting user	The requesting user thanked all participants for replying. She said that week she had been a bit worried as her symptoms were worse, though now she could cope knowing that this was the norm.	Thank you message	

**Figure 1:**
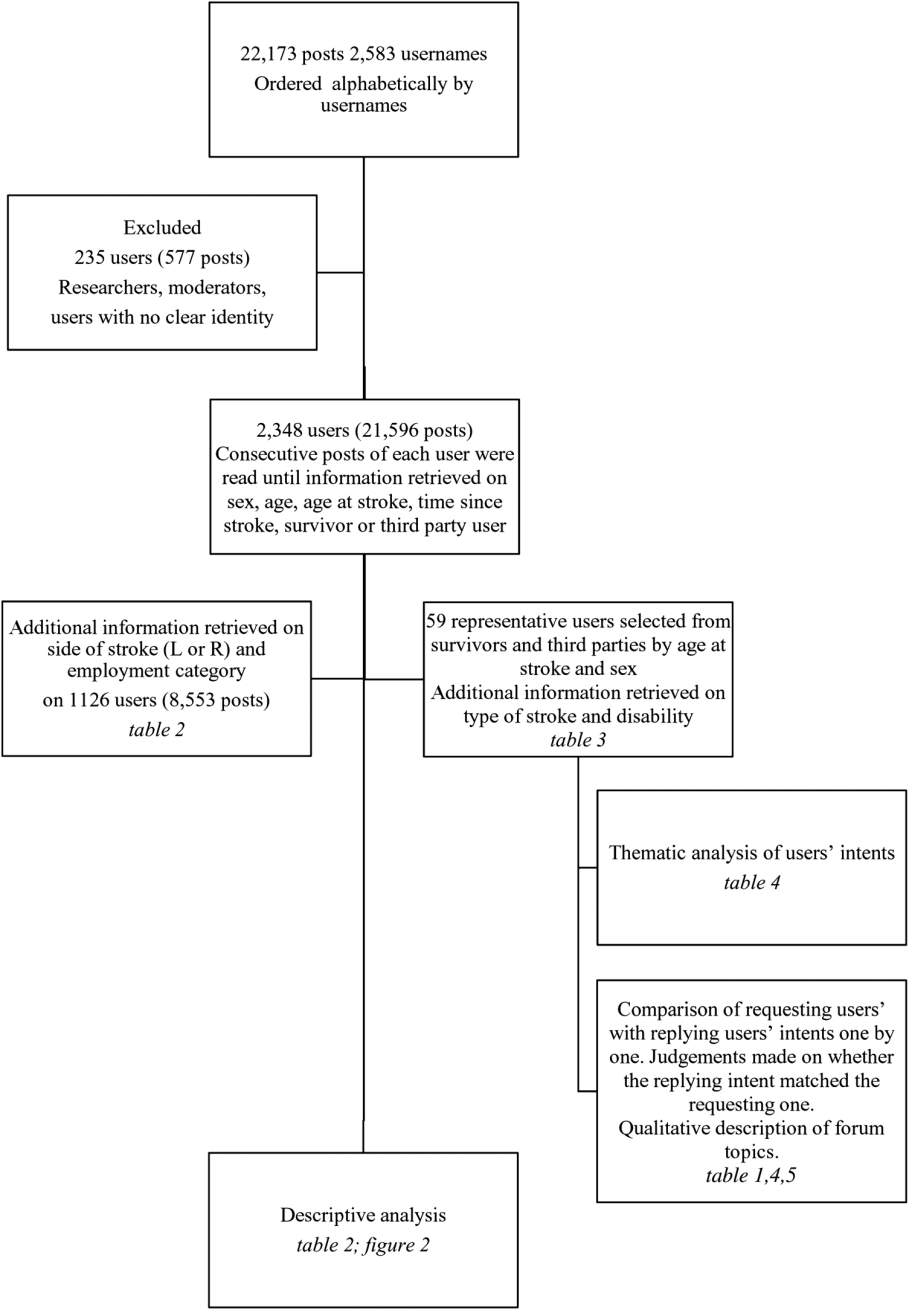
Flowchart of forum analysis.

Thematic analysis^40^ was applied to posts from a representative sample of participants in order to categorise intents behind each post and subsequent replies. The forum analysis is summarised in a flowchart (figure 1).

### Descriptive analysis of forum users' population

Username, sex, age at stroke, age at time of posting, time since stroke, whether participant was a survivor or a patient discussed by third party, third party relation with patient (eg, son, daughter, sister etc) and number of posts/participants were retrieved where available within the posts. ADS and AS collected the information on forum users. Results were subsequently compared, inter-rater agreement was 99%.

Half of users were characterised with regard to side of stroke (left or right, L/R) and employment category. The side of stroke was judged from the description of unilateral paralysis symptoms. Social class was determined using the Occupation coding tool^41^ according to the standard occupational classification 2010 (SOC2010). For the great majority of participants the occupation referred to was the one prior to stroke.

**Figure 2:**
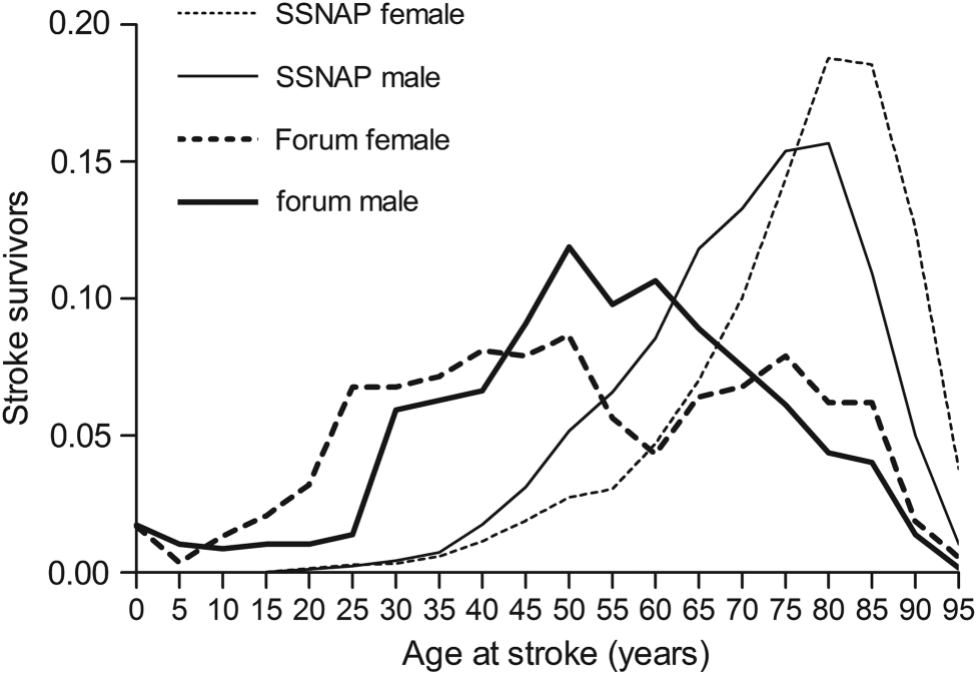
Relative frequency distributions by age at stroke and sex of forum participants and patients admitted with ischaemic stroke and primary intracerebral haemorrhage in Acute Care Hospitals in England between March 2013 and March 2014, SSNAP (Sentinel Stroke National Audit Programme).

Comparisons of forum user population with hospital admissions with stroke (ischaemic or haemorrhagic) in England was based on Sentinel Stroke National Audit Programme (SSNAP) data (figure 2).

Population data were analysed using excel. Means and SDs are reported throughout. Comparisons between groups were evaluated using unpaired t test. All p values refer to two-tailed tests. A p<0.05 was regarded as statistically significant. We used Graphpad Prism 3.03 (GraphPad, La Jolla, California, USA) for statistical analysis.

For the sake of simplicity throughout the paper patients who only suffered a transient ischaemic attack (TIA) or minor stroke are also referred to as stroke survivors/patients with stroke.

### Qualitative analysis

#### Selection of a representative sample

To produce a sample of participants that were representative of the forum population according to sex and ages at stroke, usernames were divided in two categories, stroke survivors and patients talked about by third parties. Usernames were then ordered in an excel data set according to sex and age at stroke and divided in nine groups comprising of 10 years: from 0–10 years, up to 91–100 years. Two participants were selected from the users' categories: users without the information about age at stroke and sex; male users without information about age at stroke; female users without information about age at stroke. Each group was ordered alphabetically according to usernames. The first two participants per group were selected in the age groups 41–50, 51–60, 61–70, 71–80 years, while a single user was selected from the other age groups. This resulted in 59 participants, 27 female (11 survivors, 16 patients talked about by third party), 30 male (14 survivors, 16 patients talked about by third party), two users with no information about sex (1 survivor, 1 patient talked about by third party). Posts from requesting and replying users were grouped together, resulting in 841 posts, 370 from requesting users, 471 from replying users.

Information on type of stroke (ischaemic, haemorrhagic etc) and disabilities were retrieved from the posts that originated from the subset of participants selected for qualitative thematic analysis. Disability was evaluated using criteria from the Modified Rankin Scale.^42^ Patients were classified as suffering from severe disabilities when suffering from complete hemiplegia and requiring assistance with activities of daily living. Information about patients' ability to attend to own bodily needs without assistance was not available for the majority of patients. Therefore the actual number scoring on the Rankin Scale was not performed and the classification was limited to the degree of disability, that is, severe, mild/moderate or absent. Cognitive and memory impairments were classified as non-physical impairments.

#### Thematic analysis

Qualitative thematic analysis^40^ was used to explore the users' intents behind each post and its replies. The first 10 posts of each user and all their replies were collected in an excel data set. This selection of posts could include multiple posts of the same conversation and conversations that the users had joined. When the representative user's post was actually a reply to somebody else's post, both posts were collected as well as any further post directed to the representative user. Posts from requesting and replying users were grouped together, resulting in 841 posts, 370 from requesting users, 471 from replying users. Each post was analysed to reveal users' intents for taking part in the forum, that is, the reason behind writing the post. One to four users' intents were identified within each post, yielding a total of 1379 users' intents.

Two researchers read the posts in order to become accustomed to the data set. Next, ADS generated initial codes and systematically identified and coded users' intents for requesting posts and their replies. Codes were then collated into potential themes. CBS repeated independently phases 1–3 of thematic analysis in a subset of random participants (15 participants, 206 posts), identified users' intents and classified whether or not they were addressed by responses (for description of the latter, see the next paragraph). Coding was discussed until agreement was reached, and the coding framework and coding for the other 44 participants was revised as well. The fourth phase consisted of an in-depth exploration of the detailed analysis, generating a thematic map of main themes and subthemes. In phase 5, themes were refined and the analysis finalised.

#### Evaluation of whether expressed needs were addressed by responses

Replying users' intents were compared with requesting users' intents one by one and classified according to whether or not they were accomplished (codes used: yes, not, unsure). An intent was classified as accomplished when the intent of the replying user was matching the requesting one, that is, the information or support requested were received (see table 1 as example). The code unsure was used whenever there were doubt about the reply matching the request, for example, a user asking information about the potential of eyesight recovery after stroke because of its effects on fitness to drive and the replying user empathising the lengthy process of getting his driving license back following stroke, with no mention of eyesight recovery.

The topics for which needs were expressed were classified using qualitative description.^43^

## Results

The archives comprise 2 100 053 words (equivalent of around 8000 pages of text) and 22 173 posts dated between 2004 and 2011.

### Description of forum users

The postlanguage was English. The forum included 2583 different usernames: 957 stroke survivors, 1391 third parties writing about a patient with stroke (table 2). Two-hundred and thirty participants were excluded from the analysis as they identified themselves as students or healthcare professionals asking questions for research purposes (n=43), forum moderators, or posting single short messages (n=153) from which it was not possible to gauge whether they were survivors, third party or users unrelated to stroke (figure 1). Therefore the 2348 participants included in this study were either survivors or patients with stroke talked about by third parties.

**Table 2:** Population characteristics

	Stroke survivors					Patients by third party					All				
	N	Per cent	Mean	Median	SD	N	Per cent	Mean	Median	SD	N	Per cent	Mean	Median	SD
Sex															
Females	407	*43*				598	*43*				1005	*42*			
Males	338	*35*				784	*56*				1122	*48*			
No sex info	212	*22*				9	*1*				221	*10*			
Total	957					1391					2348				
Age at stroke (years)															
Females	237	*49*	38*		12	294	*44*	63*		22	531	*46*	51		22
Males	193	*40*	44*		14	379	*56*	58*		20	572	*50*	53		19
No sex info	51	*11*	37		12	0					51	*4*	37		12
Total	481		40*		13	673		60*		21	1154		52		20
Side of stroke															
Right	104	*24*				135	*20*				239	*21*			
Left	73	*17*				181	*26*				254	*23*			
No info	264	*59*				369	*54*				633	*56*			
Total	441					685					1126				
Years since stroke															
Females	144		2	0	5	258		1	0	2	402		1.4	0	4
Males	140		2	0	4	309		0.7	0	3	449		1.1	0	3
No sex info	81		1	0	2	2					83		1	0	1
Total	365		1.8*	0	4	569		0.8*	0	3	934		1.2	0	3
N posts/participant															
Females			11	3	38			5	2	8	1004		7	2	23
Males			11	3	33			5	2	12	1221		7	2	23
No sex info			2	1	3						221		2	1	3
Total	955†		9*	3	31	1391		5*	2	10	2346		7	2	22
Social class															
Not stated	344					631					975				
Working (no info on job type)	18					16					34				
1.High managerial/professional	29	*36*				12	*31*				41	*35*			
1.1 Large employer/high managerial	*8*					*5*					*13*				
1.2 Higher professionals	*21*					*7*					*28*				
2 Lower managerial/professional	21	*26*				12	*31*				33	*29*			
3 Intermediate occupations	14	*17*				5	*13*				19	*16*			
4 Small employers/self-employed	1	*2.5*				1	*3*				2	*2*			
5Lower supervisory/technical	4	*5*				1	*3*				5	*4*			
6 Semiroutine occupations	1	*2.5*				3	*8*				4	*3*			
7 Routine occupations	9	*11*				4	*11*				13	*11*			
Total	441					685					1126				

Third party users	Female carers					Male carers					No sex info carers		All	
													N	Per cent
Sons and daughters	73	*55*				121	*64*				224	*82*	818	*59*
Partners	262	*31*				53	*28*				0	*0*	315	*23*
Grandchildren	42	*5*				8	*4*				26	*10*	76	*5*
Parents	48	*6*				0	*0*				4	*1*	52	*4*
Siblings	27	*3*				8	*4*				19	*7*	54	*4*
Relatives													38	*3*
Friends													27	*2*
Professionals													11	*1*
Total	852					190					273		1391	

*Indicates p<0.05.

†Analysis excluded two survivors (1 female and 1 male) who contributed with 4932 and 978 posts, respectively.

Information about sex was retrievable in 91% (2127) patients, while age at stroke and time since stroke was retrievable in 49% (1154) and 40% (934), respectively.

Female stroke survivors were significantly younger than males (38(13) vs 44(14) years, p<0.01), though female patients discussed by a third party were significantly older than male patients (p<0.05; table 2).

The forum included participants who suffered with stroke during the first 5 years of life (see figure 2), of which the majority were patients talked about by third party (see online supplementary figure S1A–D, in particular S1D). Despite survivors took part in the forum from a wide range of times since their stroke (mean time 1.8 years), most posts dated within a year after stroke, with an overall median time of 0 years.

10.1136/bmjopen-2015-010501.supp1 Supplementary figures

Information about side of stroke and employment category was available for a limited number of users (table 2). Twenty-four per cent of patients reported unilateral symptoms consistent with an R and 23% with L side stroke, while unilateral symptom descriptions could not be retrieved in 56%.

Information about employment was retrieved mainly from patients with class I and II employment (table 2).

Ninety seven per cent of third parties were close family members of patients with stroke they were writing about, 59% children, 23% partners, 5% grandchildren, 4% parents, 4% siblings and 3% relatives.

Analysis of a representative sample (see table 3) suggests that 76% of stroke survivors who were posting in the forum suffered from physical disabilities following an unspecified stroke. One third of users described suffering from severe disabilities, 56% cognitive and memory problems, 20% low mood and depression and 36% fatigue. Within patients described by third party, 91% suffered from physical disabilities following an unspecified stroke, of which 69% were classified as severe. Eight one per cent of patients had cognitive and memory problems, 28% low mood and depression and 9% fatigue.

**Table 3:** Type of stroke and disability degree as evaluated in the sample of 59 representatives forum participants

Sample of participants	Stroke survivors		Patients by third party		All	
	N	Per cent	N	Per cent	N	Per cent
Type of stroke						
Stroke (unspecified)	21	*78*	27	*79*	48	*81*
Ischaemic	1	*4*	2	*6*	3	*5*
Haemorrhagic	1	*4*	1	*4*	2	*3*
TIA	3	*11*	3	*9*	6	*11*
Disabilities						
Physical	19	*76*	32	*91*	51	*86*
Severe physical	7	*28*	22	*69*	29	*49*
Non-physical	14	*56*	26	*81*	40	*68*
Low mood/depression	5	*20*	9	*28*	14	*24*
Fatigue	9	*36*	3	*9*	12	*20*

The mean number of posts written by stroke survivors was about double the one by third party users (9(31) vs 5(10) posts, p<0.001).

#### Comparison of forum with SSNAP population

The population of survivors and of patients described by third party had near normal distributions, spanning all ages (figure 2).

The age range 45–65 year olds was most represented in the forum, while the age range of most hospital admissions with stroke (ischaemic or haemorrhagic) in England is 75–85 year (SSNAP data, figure 2). While hospital admissions for stroke in the 0–20 year old category are relatively rare and grouped together by SSNAP, survivors in this age group were well represented in the forum. Most children with stroke were patients talked about by third party (see online supplementary figure S2A–D, in particular S2C and S2D. It is these children that are the participants in the 0–10 age group. If an age was not available, it was coded as missing.

There was a greater difference in ages between males than females when comparing SSNAP data with forum participants.

### Thematic analysis of users' intents for posting in the forum

The main themes for posting users' intents were: request of information, requests of support, provision of information/support, sharing own experience of stroke, thank you messages (see table 3 for classification of stroke types and disabilities, and table 4 for users' intents for posting in the forum. Both table 3 and 4 summarise data from the sample of 59 participants).

**Table 4:** Number of posts for each of the main themes identified within users’ intents and classification of whether requesting users’ intents were met by the replies received

Users’ intents—themes	Intents in requesting users’ posts Number of posts *(%)*	Intents in replying users’ posts Number of posts *(%)*
Requesting information/support	215 *(58)*	32 *(7)*
Requesting support only	86 *(23)*	0 *0*
Sharing own story	223 *(60)*	267 *(56)*
Providing information/support	33 *(9)*	449 *(95)*
Thank you messages	86 *(23)*	1 *(3)*
**Intents accomplished** **N, (%)**	**Intents NOT accomplished** **N, (%)**	**Unsure** **N, (%)**
1325 *(95)*	32 *(2)*	22 *(2)*

The topics for the requests of information/support and the linked provisions of such information/support are reported in table 5, in order of frequency. Within the first 10 posts stroke survivors made contact with a mean of 7(SD 4) other participants, of whom 69% were survivors themselves. Third party users made contact with a mean of 4(SD 3) other participants, of which 57% were survivors.

**Table 5:** Forum topics. Main topics are reported in order of relevance. N of users indicates how many participants contributed to posts for each topic

Forum topics	Number of users	Number of posts	*% of all posts*
Stroke physical symptoms (communication impairments; spasms, pains, headaches; unusual symptoms; disabilities; cognition; weather effect on symptoms).	49	102	*15*
Underlying cause of stroke (undergoing diagnostic tests: Doppler, angiogram; high blood pressure, high cholesterol, migraine and PFO)	25	67	*10*
Potential for recovery (timeframe; age influence; recovery of functions: walking, upper limb movements, swallowing, reading, typing, memory, communication; doctors’ views on recovery potential)	32	59	*9*
Healthcare professionals (consultations; empathy and listening; point of contact)	18	53	*8*
Lifestyle changes (exercise; smoking cessation; alcohol)	14	49	*7*
Lack of understanding of invisible stroke impairments (family members, partners, stroke survivors themselves, doctors, general public)	8	42	*6*
Drugs and medicines (side effects; compliance, antiplatelets/anticoagulants)	18	36	*5*
Talkstroke forum (source of help, information, support, inspiration, hope)	10	36	*5*
Importance of positive attitude (facing problems day by day, laughter, patience, goal setting)	9	33	*5*
Fear and chances of stroke recurrence	13	30	*5*
Sources of help, information, support (Internet, stroke charities, local stroke clubs, family, friends)	24	24	*4*
Post-stroke fatigue	5	22	*3*
Holidays, travel and travel insurances	5	17	*3*
Work after stroke, benefits	6	16	*2*
Driving (getting driving licence back, driving, car insurance, car adaptation)	4	14	*2*
Personality change and mood swings	5	14	*2*
Carers issues	12	12	*2*
Depression and anxiety	10	11	*2*
Emotional lability	3	10	*2*
Stroke in children	4	9	*1*
Post-stroke epilepsy	2	6	*1*

PFO, patent foramen ovale.

### Forum users received the information and support requested

A total of 1379 users' intents were identified within the 841 requesting and replying posts. Ninety-five per cent (1325) of intents were met (see table 1 and table 4).

Of note there were occasions where the replying posts were offering information of greater value than originally requested, for example, appropriate advice to seek urgent medical attention for symptoms suggestive of serious conditions or to check potential interactions of medications; suggestions of consulting health services about symptoms otherwise considered incurable, for example, pains and spasms.

Whenever inappropriate medical information or health behaviour were brought up (eg, stopping a medication due to side effects), they were promptly and appropriately counteracted by forum users (eg, advised to seek healthcare advice).

Only 9 of 370 requesting posts (2%) did not receive replies.

#### Forum topics

Twenty-one main topics were identified from the 841 posts from the representative sample (see table 5), that is, 4% (841/22 173) of the total posts in the forum archive.

Stroke-related impairments, finding out the cause of the stroke and the potential for recovery were the main topics discussed. Healthcare professionals and medicines were important participants of conversations as well as fear and likelihood of stroke recurrence. The lack of understanding of stroke effects, especially the invisible effects, such as fatigue, emotional lability, impaired self-confidence, and memory and cognitive problems, was the topic generating the highest number of posts/user. Nearly half of participants wrote posts about sources of information and support they had found helpful. Fatigue, fear of stroke recurrence and emotional lability were reported in multiple posts by the same users, as well as the importance of a positive attitude during recovery. The forum itself was often mentioned and described as a key source of information, support, help but also hope and inspiration.

Topics were discussed in a timeframe that spanned from the initial acute hospital admission with stroke, to the rehabilitation phase, up to long-term recovery from stroke.

Needs of information and support expressed by users were experienced at the time the messages were written, and contained detailed and vivid descriptions of personal stories.

## Discussion

### Principal findings

This study shows that stroke survivors of both sexes and across a range of ages, stroke type and disabilities were active users of an online forum, although generally representing a younger population than those admitted to hospital. Survivors and third parties were together taking part in the discussions. Severe disability did not preclude access to the forum. The forum was mainly used as a means to ask and receive information and support and sharing one's own story of stroke. The great majority of users' intents were met by the replies received and a wide variety of topics were discussed. Information and support were provided on an individual basis and contextualised with personal experiences from requesting and replying users. Online forums for patients with stroke represent a potential resource for studying unmet needs in this population, complementing the evidence from traditional research studies, as shown in a sister paper.^44^

### Strengths and limitations

The strength of the study lays in the descriptive analysis of the entire population of 2348 forum participants. The characterisation of sex, ages at stroke, stroke types and disabilities underpins the validity of results obtained from analysing the forum archives. Moreover, it ensures the appropriateness of the selection of the representative sample of participants to study users' intents and topics of discussion. Forum discussions are self-initiated and people communicate with each other without time, length or behavioural constraints, offering a window to understand patients' issues that by-pass reactivity and self-representation bias of traditional research approaches. Additionally, forums might include people who do not take part in traditional research studies, therefore offering perspectives from an unrepresented patient population.^45^ Forum participants were a younger population compared with patients with stroke from SSNAP. Considering they represent a prevalent population compared with the incident population admitted with stroke (SSNAP data), the discrepancy in age with a representative population would be larger. This might be down to younger stroke survivors being more aware of what their unmet needs are and accessing an online forum as a resource for such needs.^46^ The analysis of the forum was limited by the time users were active in the forum and the amount of information exchanged, for example, participants might have experienced additional impairments not mentioned in the forum, making the evaluation of disability not comprehensive. The forum was moderated and some of the posts might have been removed or affected by the moderation process. The authenticity of the forum content could not be assessed.

The process of retrieving information on the 2500 forum participants was time consuming. Linguistic analysis tools currently available do not allow extracting reliably information about characteristics of participants like sex, age and disability, nor users' intents for taking part in an online forum. There is a need to develop appropriate and efficient analytical methods to study large volumes of text.^29^

Finally, the population of users included non-active users, that is, participants who were reading posts without taking part in the discussions. Although such users could be more numerous than the actual registered ones, this population could not be quantified nor characterised. A study reported 26:1 lurkers for every author of an online forum message.^30^

### Characteristics of patients with stroke associated with participating in an online stroke forum

Male and female stroke survivors took part in the forum (figure 2), outlining the relevance of this online service for both sexes, albeit the evidence that the female gender^47^ ^48^ is associated with more frequent health-related Internet use.^49^

Non-physical disability, fatigue and depression were relatively common amongst stroke survivors using the forum. This is in keeping with other studies of stroke sequelae,^32^ ^50–52^ suggesting that disability did not preclude access to an online forum.

Although 957 (41%) of participants were stroke survivors who were younger and potentially more cognitively sound and verbal about their experience than the population of patients with stroke, the majority of participants (1391, ie, 59%) were patients with stroke talked about by third party, therefore potentially representing the population of stroke sufferers less able to communicate via computers and more disabled. Indeed evaluation of disability from the sample of participants showed that 91% of patients discussed by third party were suffering from physical disability, which was severe in 59%. This is in contrast with users who were stroke survivors themselves, 76% of which were suffering from physical disability, and only 28% from severe physical disability. Interestingly, non-physical disability like cognitive and memory impairments were more prevalent amongst stroke survivors (56%) than in patients with stroke discussed by third party (40%).

Although all employment classes were described, higher classes were over-represented. This could be down to either over-representation of users with higher social classes or to people with class I and II employment being more likely to talk about their job during discussions. This is consistent with previous research linking higher education and income with frequent health-related Internet use.^48^ Given that employment was not disclosed by the great majority of participants, it was not possible, though, to draw conclusions on how participants' social classes compared with the overall population of patients with stroke.

More third party users than survivors took part in the forum discussion, though survivors posted twice as many messages. This is in contrast to other online forums where users are mainly patients themselves.^25–28^ Most third party users provided information about their sex, revealing a ratio of 4:1 of female compared to male carers, confirming the predominance of female gender in caregiving.^53^ Third party users were actively taking part in the forum in the first year after stroke, and in particular represented patients with more severe disability. This might be down to more unmet needs of information and support in the first period after stroke in patients with severe disability and their families, and/or younger family members/carers considering online forum as potential source of advice.

### Implications for clinicians and future research

Unmet needs were reported across a range of clinical domains, including mobility and communication, recovery potential, interaction with healthcare professionals, medicines and emotional well-being as well as need for information and aspects of social participation. Although forum participants were a ‘young’ population, they still raised the same issues as have been identified in research on more representative populations.^1^ ^3^ ^46^ A survey study to investigate unmet needs that included patients of older ages did not find differences in the range or type of unmet need reported by age category, suggesting that age per se may not be associated with perception of unmet need.^1^

Most posting users' intents were accomplished by replies received. Forum users provided help and support on an individual basis and contextualised with their own personal stroke experiences. This suggests that the information and support were provided on an individual basis, which is preferable to patients and their family carers.^54^

A recent systematic review showed that social networking interventions have a positive effect on health behaviour-related outcomes,^55^ and a before and after study suggests benefit of an Internet support forum in people with dementia.^56^ Through the forum stroke survivors and their families benefited from mutual emotional and practical support, social participation, education, introduction to available resources. The forum could therefore be considered in terms of a self-management tool run by survivors and family carers, according to the holistic definition of self-management proposed by the US Institute of Medicine “the tasks that individuals must undertake to live with one or more chronic conditions. These tasks include having the confidence to deal with medical management, role management and emotional management of their conditions”.^57^ ^58^ The value of the service provided by the forum was reflected in the number of posts about the forum itself (5% of posts) and the high percentage of posts that were or included thank you messages (23% of posts from requesting users). Inappropriate medical information or health behaviours were identified and corrected by participants in subsequent postings. This is consistent with analysis of online support groups for patients with cancer.^59^

Future studies should establish whether access to an online forum translates into improving health outcomes after stroke. The relatively low costs of providing and maintaining an online forum service and the voluntary basis of its participants' contribution are likely to make such an intervention cost-effective.
