# Supplementary material for: Stroke survivors and their families receive information and support on an individual basis from an online forum: descriptive analysis of a population of 2348 patients and qualitative study of a sample of participants
Source: BMJ Open. 2016 Apr 5;6(4):e010501. doi: 10.1136/bmjopen-2015-010501 (PMC4823439; doi:10.1136/bmjopen-2015-010501)

Supplementary Figures:

Figure S1. Frequency distribution of forum users by sex, age at stroke and participants survivors versus patients with stroke talked about by third party.

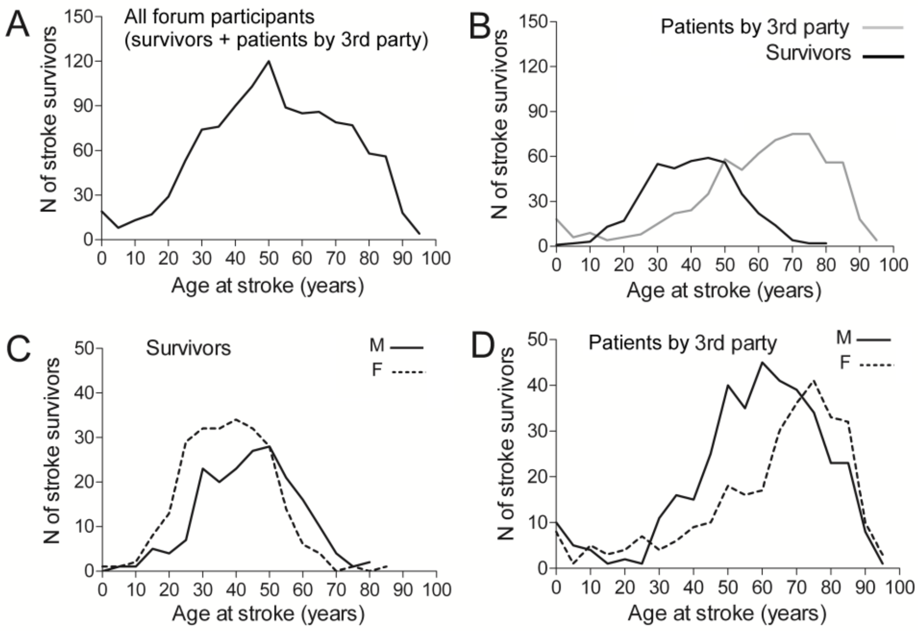

Figure S2. Relative frequency distributions by sex, age at stroke, survivors/ patients-with stroke-talkedabout-by-third-party versus patients admitted with ischaemic stroke and primary intracerebral haemorrhage in Acute Care Hospitals in England between March 2013 and March 2014 , SSNAP (Sentinel Stroke National Audit Programme). Black and white bars represent males and females, respectively.

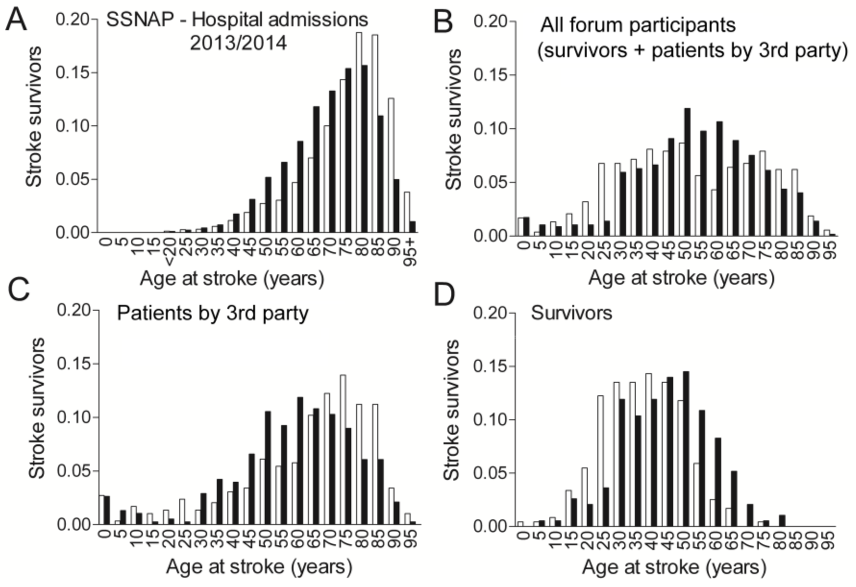

Supplement: Supplementary figures [file bmjopen-2015-010501supp_figures.pdf]
